# Predictors of unfavourable outcome in adults with suspected central nervous system infections: a prospective cohort study

**DOI:** 10.1038/s41598-023-48472-z

**Published:** 2023-12-01

**Authors:** Liora ter Horst, Ingeborg E. van Zeggeren, Sabine E. Olie, I-PACE Study Group, J. Brenner, J. Citroen, B.M. van Geel, S.G.B. Heckenberg, K. Jellema, M.I. Kester, J. Killestein, B.B. Mook, Y.C. Resok, M.J. Titulaer, K.E.B. van Veen, C.V.M. Verschuur, Diederik van de Beek, Matthijs C. Brouwer

**Affiliations:** 1 Amsterdam UMC, Department of Neurology, Amsterdam Neuroscience, University of Amsterdam, Meibergdreef 9, PO Box 22660, 1105 AZ Amsterdam, The Netherlands; 2 Amsterdam UMC, Amsterdam, The Netherlands; 3 Erasmus MC, Rotterdam, The Netherlands; 4 Onze Lieve Vrouwe Gasthuis, Amsterdam, The Netherlands; 5 Noordwest Ziekenhuisgroep, Alkmaar, The Netherlands; 6 Spaarne Gasthuis, Haarlem, The Netherlands; 7 Haaglanden MC, Den Haag, The Netherlands; 8 Flevoziekenhuis, Almere, The Netherlands; 9 Haga Ziekenhuis, Den Haag, The Netherlands; 10 Alrijne Ziekenhuis, Leiden, The Netherlands; 11 Albert Schweitzer ziekenhuis, Dordrecht, The Netherlands

**Keywords:** Central nervous system infections, Meningitis, Outcomes research, Neurology

## Abstract

Suspected central nervous system (CNS) infections may pose a diagnostic challenge, and often concern severely ill patients. We aim to identify predictors of unfavourable outcome to prioritize diagnostics and treatment improvements. Unfavourable outcome was assessed on the Glasgow Outcome Scale at hospital discharge, defined by a score of 1 to 4. Of the 1152 episodes with suspected CNS infection, from two Dutch prospective cohorts, the median age was 54 (IQR 37–67), and 563 episodes (49%) occurred in women. The final diagnoses were categorized as CNS infection (N = 358 episodes, 31%), CNS inflammatory disease (N = 113, 10%), non-infectious non-inflammatory neurological disorder (N = 388, 34%), non-neurological infection (N = 252, 22%), and other systemic disorder (N = 41, 4%). Unfavourable outcome occurred in 412 of 1152 (36%), and 99 died (9%). Predictors for unfavourable outcomes included advanced age, absence of headache, tachycardia, altered mental state, focal cerebral deficits, cranial nerve palsies, low thrombocytes, high CSF protein, and the final diagnosis of CNS inflammatory disease (odds ratio 4.5 [95% confidence interval 1.5–12.6]). Episodes suspected of having a CNS infection face high risk of experiencing unfavourable outcome, stressing the urgent need for rapid and accurate diagnostics. Amongst the suspected CNS infection group, those diagnosed with CNS inflammatory disease have the highest risk.

## Introduction

Patients suspected of a central nervous system (CNS) infection often present with severe illness, including decreased consciousness, neurological deficits and hemodynamic instability^1^. Diagnosing these patients frequently poses a challenge due to the wide range of possible conditions, ranging from life-threatening diseases such as bacterial meningitis or septic encephalopathy to more benign and sometimes self-limiting diseases such as migraine or systemic viral infections^1^. Previous studies have reported an overall mortality of 10% and incomplete recovery in an additional 17% in this population^1,2^. Prompt diagnostic work-up, identification of the cause-specific diagnosis, and early targeted treatment have been shown to be crucial in improving outcome, particularly in patients with bacterial meningitis^2–7^. However, clinical characteristics and ancillary investigations often lack sensitivity and/or specificity to differentiate between these various causes, although cerebrospinal fluid (CSF) leukocyte count differentiated best between bacterial meningitis and other diagnoses in this population^1^. Difficulty in making the diagnosis may lead to delayed or unnecessary treatment with antibiotics and antiviral drugs. To improve outcome in this patient population, it is essential to recognize high-risk categories for unfavourable outcome. This prospective study aims to determine predictors for an unfavourable outcome to identify subgroups for enhancing diagnostics and treatment.

## Methods

### Patient inclusion and data collection

We included episodes from two prospective cohort studies performed between 2012 and 2015 and between 2017 and 2022. The first study (PACEM—Paediatrics and Adult Causes of Encephalitis and Meningitis) was a single-centre study, and a pilot study for the second study (I-PACE—Improving Prognosis by using innovative methods to diAgnose Causes of Encephalitis), which is an ongoing multi-centre study running in 11 Dutch hospitals^1^. Both studies included adult patients aged 16 years or older with suspected CNS infection presenting to the emergency department or inpatients who underwent CSF examination. Episodes were identified during morning rounds or reported to the investigators by the treating physician. Physicians could contact the investigators 24/7 to include patients. Episodes of suspected CNS infections within three months after head trauma or neurosurgery, and those with a neurosurgical device in situ, were excluded.

Data on patient characteristics, medical history, symptoms and signs on admission, laboratory results, radiological examination, treatment and outcome were collected in online case record forms. All patients and/or their legal representatives have given written informed consent for this study after receiving written information about the study. All patient data were rendered anonymous, and the study was carried out in accordance with Dutch privacy legislation.

### Procedures and definitions

Episodes were classified as suspected nosocomial CNS infection if the suspicion occurred during hospital admission (> 48 h after presentation) or within one week after discharge^8,9^. All other episodes were classified as community-acquired. Neurological examination was performed upon admission and at discharge. The level of consciousness was scored using the Glasgow Coma Scale (GCS)^10^. An altered mental state was defined as a GCS score of < 14 and coma as a GCS score of ≤ 8. Patients were considered immunocompromised if they were using immunosuppressive drugs or had a medical history of diabetes mellitus, alcoholism, HIV infection or a splenectomy.

Outcome at discharge was scored according to the Glasgow Outcome Scale (GOS), a well-validated scale ranging from 1 to 5. A score of 1 indicates death, 2 vegetative survival, 3 severe disability, 4 moderate disability, and 5 indicates mild or no disability^10^. A score of 5 was considered a favourable outcome. If pre-existing conditions were the cause of the outcome score below 5 on the GOS, and the patient’s condition did not worsen due to the current episode, we classified the outcome as favourable.

### Diagnostic categorization

The final diagnosis of all included episodes was classified into five categories, as previously described^1^. The categories were; (1) CNS infection, (2) CNS inflammatory disease, (3) non-infectious non-inflammatory neurological disorder, (4) non-neurological infection, and (5) other systemic disorders. Two clinicians independently classified the final diagnoses in the five categories based on all available clinical, laboratory and follow-up data. If there was no consensus, a third investigator was consulted. Inter-rater agreement between the first assessors was assessed by calculating the kappa coefficient, which was 0.76 in cohort 1 and 0.64 in cohort 2.

### Statistical analysis

Statistical analyses were conducted using SPSS statistical software, version 28 (SPSS Inc.) and R studio version 4.0.3. We used descriptive statistics for baseline characteristics, with medians and interquartile range (IQR, describing their 25th to 75th percentile). Comparisons were made with the Mann–Whitney U test used for continuous data, and the Fisher exact test was used for categorical data. All tests were 2-tailed, and *P* < 0.05 was considered significant. We chose possible predictors of an unfavourable outcome based on previous research and availability to examine the predictor early upon disease presentation^11^. We investigated the association between these predictors and outcomes with logistic regression, providing odds ratios (ORs) and 95% CIs. Univariable and multivariable binary logistic regression models assessed prognostic factors for discharge outcomes. For these multivariable logistic models, missing values in the selected prognostic factors were imputed (median 2.1% per prognostic factor [IQR 0.33–8.8%]). Non-normally distributed continuous variables were transformed into categorical variables.

### Standard protocol approvals, registrations and patient consents

The two studies were approved by the Biobank Ethics Assessment Committee of the Amsterdam UMC; number AMC 2014_290. Written informed consent was obtained from all participants or their representatives. All methods were performed in accordance with this approval.

## Results

**Figure 1:**
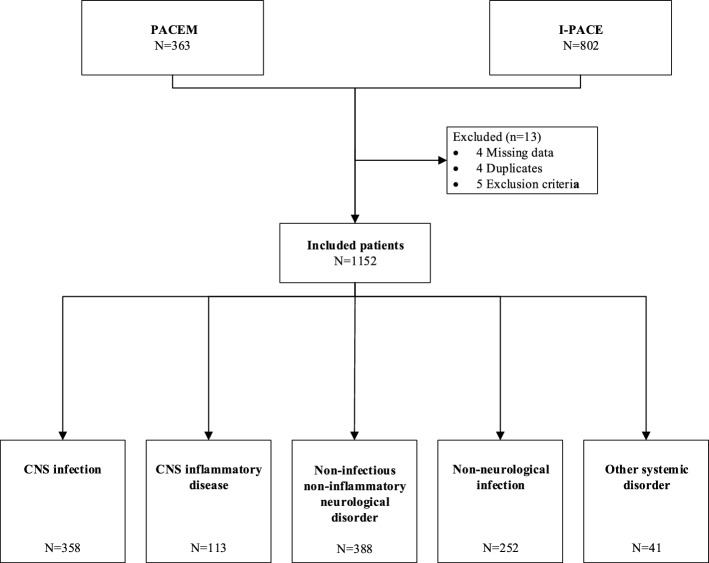
Selection of patients.

A total of 1165 episodes were included: 363 episodes in the PACEM study and 802 in the I-PACE study. Of these, 13 episodes (1%) were excluded based on exclusion criteria or missing outcome data (Fig. 1), resulting in 1152 episodes in 1127 patients. Patients were evaluated at the emergency department in 861 of 1140 episodes (76%), at the intensive care in 59 (5%), and 220 (19%) at other clinical departments. The episode was classified as nosocomial in 106 of 1137 (9%)^8,9^.

The median age was 54 years (IQR 37–67), and 563 episodes (49%) occurred in women (Table 1). An immunocompromising condition was present in 450 of 1151 episodes (39%), which was due to HIV infection in 74 of 1150 (6%), the use of immunosuppressive drugs in 208 of 1149 (18%), and diabetes mellitus in 188 of 1151 (16%; Table 1). In 417 of 1111 episodes (38%), symptoms were present for less than 24 h. The most common symptoms included headache in 639 of 998 episodes (64%), fever in 466 of 1051 episodes (44%), and neck stiffness in 188 of 892 episodes (21%). An altered mental state was present in 364 of 1143 episodes (32%) and neurological deficits in 347 of 754 (46%).

**Table 1:** Characteristics of all episodes with Suspected Central Nervous System infections (n = 1152).

	1152 patients	Characteristic	1152 patients
Age	54.0 (37–67)	Heart rate	90 (76–105)
Female sex	563/1152 (49)	Diastolic blood pressure	78 (68–89)
**Medical history**		Aphasia or Paresis	249/896 (28)
Immunocompromised state	450 /1151 (39)	Seizures on admission	159/1078 (15)
HIV	74/1150 (6)	Cranial nerve palsy	155/1052 (15)
Splenectomy	6/1148 (1)	**Laboratory results**	
Immunosuppressive treatment	208/1149 (18)	Thrombocytes × 10^12^/L	230 (168–287)
Diabetes	188/1151 (16)	C-reactive protein mg/L	16 (3–70)
Alcoholism	62/1023 (6)	Blood leukocyte count × 10^9^/L	9.3 (6.6–13.2)
Other focus of infection*	112/1152 (10)	CSF leukocytes /mm^3^	5 (1–60)
**Symptoms on presentation**		CSF leukocytes ≥ 4 cells/mm^3^	517/1139 (45)
**Location of neurology presentation**		CSF protein < 0.60	463/1149 (40)
Emergency department	861/1140 (76)	**Glasgow Outcome Scale score**	
Inpatient departments	220/1140 (19)	1—Dead	99/1152 (9)
Intensive care unit	59/1140 (5)	2—Vegetative survival	2/1152 (0.2)
Symptoms < 24 h	417/1111 (38)	3—Severely disabled	91/1152 (8)
**Glasgow Coma Scale score**		4—Moderately disabled	220/1152 (19)
Median (IQR)	15 (13–15)	5—Good recovery	740/1152 (64)
GCS < 14	364/1143 (32)		
GCS ≤ 8	123/1143 (11)		
Neck stiffness	188/892 (21)		
Headache	639/998 (64)		
Temperature ≥ 38.5˚C	466/1051 (44)		

Data are median (IQR) or n/N (%). Abbreviations: GCS = Glasgow Coma Scale; CSF = cerebrospinal fluid; DBP = diastolic blood pressure. *Otitis and/or sinusitis and/or pneumonia. ^a^Age known in all episodes. ^b^ Glasgow Coma Scale score was known for 1143 episodes. ^c^Heart rate was known for 1112 episodes. ^d^Diastolic blood pressure was known for 1117 episodes. ^e^Thrombocytes was known for 1094 episodes. ^f^C-reactive protein was known for 1039 episodes. ^g^Blood leukocyte count was known for 1119 episodes. ^h^CSF leukocyte count was known for 1139 episodes.

A lumbar puncture was performed in all episodes, and CSF examination showed an elevated leukocyte count (≥ 4 cells/mm^3^) in 622 of 1139 episodes (55%). The CSF leukocyte count was between 4 and 99 cells/mm^3^ in 378 (33%) episodes, between 100 and 999 cells/mm in 147 (13%), and more than 1000 cells/mm^3^ in 97 (9%) episodes. During the clinical course, antibiotics according to bacterial meningitis regime or antiviral treatment were started in 695 of 1150 episodes (60%).

A final clinical diagnosis was available for all episodes. CNS infection was diagnosed in 358 (31%), CNS inflammatory disease in 113 (10%), non-infectious non-inflammatory neurological disorder in 388 (34%), non-neurological infection in 252 (22%), and other systemic disorder in 41 (4%, Table 2). Of the 358 CNS infections, the diagnosis was microbiologically confirmed in 236 episodes (66%). CSF culture was positive in 79 of 236 (33%) episodes, CSF PCR in 117 (50%), CSF antigen testing in 19 (5%), blood culture in 92 episodes (39%), and blood PCR in 17 (7%).

**Table 2:** Final diagnoses in 1152 episodes.

	Number of episodes N = 1152	Unfavourable outcome N = 412	Favourable outcome N = 740	P-value
**Central Nervous System Infection**	358 (31)	118/358 (33)	240/358 (67)	
Bacterial meningitis	138/358 (39)	51/138 (37)	87/138 (63)	
Viral meningitis	108/358 (30)	11/108 (10)	97/108 (89)	
Viral encephalitis	54/358 (15)	32/54 (59)	22/54 (41)	
Other CNS infections	58/358 (16)	24/58 (41)	34/58 (59)	
**Central Nervous Inflammatory Disease**	113 (10)	74/113 (65)	39/113 (34)	< 0.001
Confirmed Autoimmune Encephalitis	10/113 (9)	8/10 (80)	2/10 (20)	
Paraneoplastic encephalitis	2/113 (2)	1/412 (0)	1/740 (0)	
AIE of unknown cause	26/113 (23)	18/26 (69)	8/26 (31)	
Myelitis/myelopathy	9/113 (9)	8/9 (89)	1/9 (11)	
Chronic meningitis	16/113 (14)	4/16 (25)	12/16 (75)	
Inflammatory polyneuropathy	3/113 (3)	3 (100)	0 (0)	
HaNDL syndrome	4/113 (4)	0 (0)	4 (100)	
Other CNS autoimmune diseases *	43/113 (37)	32 (74)	11 (26)	
**Non-neurological infection**	252 (22)	54/252 (21)	198/252 (79)	< 0.001
**Non-infectious non-inflammatory -neurological disorder**	388 (34)	10/388 (39)	238/388 (61)	0.15
**Other systemic disorder**	41 (4)	16/41 (39)	25/41 (61)	0.74

Data are in n/N (%). HaNDL = Headache with neurological deficits and CSF lymphocytosis; CNS = Central Nervous System. *Other CNS autoimmune diseases; Guillain-Barré Syndrome (10), Vasculitis (10), Neurosarcoidosis (9), Acute disseminated encephalomyelitis (4), Other CN autoimmune disease of unknown cause (7), Neuro SLE (2), Immune Reconstitution Inflammatory Syndrome (1).

The outcome was unfavourable in 412 episodes (36%), and in 99 of 1152 episodes (9%), the patient died (Table 3). Neurological sequelae were present in 352 of 1015 (35%) surviving patients. The rate of unfavourable outcome varied per disease category and was 118 out of 358 episodes (33%) diagnosed with CNS infections, 74 out of 113 (65%) with CNS inflammatory diseases, 150 out of 388 (39%) with non-infectious non-inflammatory neurological disorders, 54 out of 252 (21%) with non-neurological infections, and in 16 out of 41 (39%) with other systemic disorders (Table 2). The mortality rate was 36 of 358 (10%) in episodes with CNS infections, eight out of 113 (7%) with CNS inflammatory disease, 28 out of 388 (7%) with non-infectious non-inflammatory neurological disorders, 25 out of 252 (10%) with non-neurological infections two of 41 (5%) with other systemic disorders. To analyse changes in time period and outcome between cohort 1 and cohort 2, we found an unfavourable outcome in 91 of 363 episodes (25%) in cohort 1 versus 321 of 793 episodes (41%) in cohort 2, *P* < 0.001.

**Table 3:** Clinical characteristics and outcome.

	Outcome		*P*-value
	Unfavourable 412 patients	Favourable 740 patients	
Age, median	62 (49–72)	48 (32–63)	< 0.001
Female Sex	181/412 (44)	382/740 (52)	0.007
**Predisposing factors**			
Immunocompromised state	179/412 (43)	271/739 (37)	0.01
HIV	26/411 (6)	48/739 (7)	0.51
Immunosuppressive therapy	82/410 (20)	126/738 (17)	0.12
Diabetes	85/412 (21)	103/739 (14)	0.002
Alcoholism	26/364 (7)	36/659 (6)	0.17
**Symptoms on presentation**			
Symptoms < 24 h	129/385 (34)	288/726 (40)	0.03
GCS < 14	176/408 (43)	188/735 (26)	< 0.001
GCS < 8	76/408 (19)	47/735 (6)	< 0.001
Neck stiffness	62/274 (23)	126/618 (20)	0.25
Headache	132/315 (42)	507/683 (74)	< 0.001
Fever > 38 ˚C	119/398 (30)	291/719 (41)	< 0.001
Tachycardia > 120 beats/min	43/399 (11)	48/713 (7)	0.01
Diastolic blood pressure, mmhg	80 (69–92)	77 (67–88)	0.01
Aphasia or Paresis	140/346 (41)	109/702 (16)	< 0.001
Seizures on admission	74/376 (20)	85/702 (12)	< 0.001
Cranial nerve palsy	90/355 (25)	65/697 (9)	< 0.001
Thrombocytes × 10^12^/L	233 (160–301)	230 (175–283)	0.97
C-reactive protein > 40 mg/L	120/342 (35)	241/697 (35)	< 0.001
CSF leukocytes, cells/mm3	6 (2–59)	4 (1–61)	0.13
CSF leukocytes ≥ 4 cells/mm3	242/407 (60)	380/732 (52)	0.008
CSF protein > 0.6	210/408 (52)	257/733 (35)	< 0.001

Data are median (IQR) or n/N (%). Abbreviations: GCS = Glasgow Coma Scale, CSF = cerebrospinal fluid.

^a^Age was known in all episodes. ^b^Glasgow Coma Scale score was known for 1143 episodes. ^c^Diastolic blood pressure was known for 1117 episodes. ^d^Thrombocytes was known for 1094 episodes. ^e^CSF leukocyte count was known for 1139 episodes.

In the multivariable analysis, predictors for unfavourable outcome were advanced age, the absence of headache, tachycardia, GCS score < 14, focal cerebral deficits (aphasia or paresis), cranial nerve palsies, thrombocyte count < 150 × 10^12^/L, CSF protein count > 0.60 g/L, and the final diagnosis of a CNS inflammatory disease (Table 4).

**Table 4:** Predictive characteristics for unfavourable outcome.

Characteristic	Univariable OR	Multivariable OR	*P*-value
	(95% CI)	(95% CI)	
Age 16 to 39	Reference	Reference	
Age 40 to 70	3.28 (2.34–4.59)	2.01 (1.35–2.99)	< 0.001
Age > 70	6.67 (4.48–9.93)	3.46 (2.14–5.59)	< 0.001
Female sex	0.74 (0.58–0.94)	0.95 (0.71–1.28)	0.74
**Predisposing factors**			
Immunocompromised state	1.33 (1.04–1.70)	0.99 (0.73–1.34)	0.93
Other focus of infection*	0.99 (0.66–1.50)	–	
**Symptoms on presentation**			
Symptoms < 24 h	0.78 (0.60–1.01)	–	
GCS score	0.87 (0.83–0.90)	0.94 (0.89–1.00)	0.04
Neck stiffness	1.08 (0.73–1.60)	–	
Headache	0.24 (0.18–0.33)	0.39 (0.27–0.57)	< 0.001
Tachycardia > 120 beats/min	1.68 (1.09–2.58)	1.89 (1.08–3.32)	0.03
Fever ≥ 38˚C	0.62 (0.48–0.80)	0.81 (0.58–1.14)	0.23
Diastolic blood pressure < 60 mmhg	1.29 (0.85–1.95)	1.29 (0.78–2.14)	0.33
Diastolic blood pressure 60–80 mmhg	Reference	Reference	
Diastolic blood pressure > 80 mmhg	1.56 (1.20–2.02)	1.33 (0.97–1.80)	0.07
Aphasia or Paresis	3.84 (2.83–5.21)	2.01 (1.32–3.04)	
Seizures on admission	1.75 (1.26–2.45)	0.80 (0.51–1.25)	
Cranial nerve palsy	3.11 (2.17–4.46)	2.24 (1.48–3.38)	< 0.001
Thrombocytes < 150 × 10^12^/L	1.42 (1.04–1.94)	1.69 (1.15–2.47)	0.008
Thrombocytes 150 to 450 × 10^12^/L	Reference	Reference	
Thrombocytes > 450 × 10^12^/L	1.98 (0.98–4.01)	1.46 (0.67–3.20)	0.34
CRP < 40 mg/dL	Reference	–	
CRP 40–150 mg/dL	0.93 (0.64–1.35)	–	
CRP > 150 mg/dL	1.35 (0.94–1.94)	–	
Blood leukocytosis **	1.01 (0.79–1.31)	–	
CSF leukocytes < 4 cells/mm3	Reference	Reference	
CSF leukocytes 4 to 100 cells/mm3	1.54 (1.17–2.02)	1.13 (0.78–1.65)	0.53
CSF leukocytes 100 to 1000 cells/mm3	1.21 (0.82–1.77)	1.39 (0.74–2.61)	0.31
CSF leukocytes > 1000 cells/mm3	0.89 (0.56–1.43)	0.70 (0.33–1.50)	0.36
CSF protein > 0.60 g/dL	1.97 (1.54–2.53)	1.57 (1.08–2.29)	0.02
**Final diagnosis**			
CNS infection	Reference	Reference	
CNS inflammatory disease	3.98 (2.54–6.23)	3.97 (2.28–6.93)	< 0.001
Systemic infection	0.57 (0.39–0.83)	0.55 (0.31–1.00)	0.05
Other neurological disease	1.28 (0.95–1.74)	1.03 (0.64–1.68)	0.90
Non-neurological non-infectious disease	1.31 (0.67–2.54)	1.32 (0.56–3.15)	0.53

The multivariable analysis used an imputed dataset with 5 imputation rounds**,** all variables in the table were entered in the multivariable logistic regression model simultaneously. Abbreviations: GCS = Glasgow Coma Scale; CRP = C-reactive protein; CSF = Cerebrospinal fluid; CNS = Central Nervous System.

Predictors for death were advanced age (> 70 years old), an immunocompromised state, GCS score < 14, the absence of headache, diastolic blood pressure < 60 mm Hg, thrombocyte count < 150 × 10^12^/L, CRP of 40 to 150 mg/dL, and CSF protein concentration > 0.60 g/L (Table 5).

**Table 5:** Predictive characteristics for mortality.

Characteristics	Univariable OR (95% CI)	Multivariable OR (95% CI)	*P*-value
Age 16 to 39	Reference	Reference	Reference
Age 40 to 70	2.53 (1.26–5.08)	1.30 (0.57–2.93)	0.53
Age > 70	7.99 (3.92–16.3)	3.40 (1.44–8.06)	0.005
Female sex	0.79 (0.52–1.19)	–	
**Predisposing factors**			
Immunocompromised state	2.39 (1.56–3.65)	1.91 (1.16–3.16)	0.01
Other focus of infection*	2.45 (1.42–4.22)	1.51 (0.76–3.00)	0.24
**Symptoms on presentation**			
Symptoms < 24 h	1.21 (0.77–1.89)	-	
GCS score	0.80 (0.76–0.84)	0.86 (0.80–0.93)	< 0.001
Neck stiffness	1.33 (0.75–2.34)	–	
Headache	0.24 (0.14–0.43)	0.50 (0.27–0.94	0.03
Tachycardia	3.85 (2.25–6.59)	1.73 (0.87–3.43)	0.12
Fever	0.1.04 (0.68–1.59)	–	
Diastolic blood pressure < 60 mmhg	2.65 (1.49–4.71)	2.22 (1.12–4.40)	0.02
Diastolic blood pressure 60–80 mmhg	Reference	Reference	Reference
Diastolic blood pressure > 80 mmhg	1.07 (0.67–1.70)	1.17 (0.69–1.99)	0.56
Aphasia or Paresis	3.88 (2.18–6.92)	1.43 (0.80–2.56)	0.23
Seizures on admission	1.68 (1.003–2.82)	0.78 (0.41–1.51)	0.47
Cranial nerve palsy	2.21 (1.28–3.81)	1.72 (0.93–3.17)	0.08
Thrombocytes < 150	2.42 (1.51–3.87)	1.88 (1.06–3.34)	0.03
Thrombocytes 150 to 450	Reference	Reference	Reference
Thrombocytes > 450	4.06 (1.77–9.29	2.85 (1.00–8.14)	0.05
CRP < 40 mg/dL	Reference	Reference	Reference
CRP 40–150 mg/dL	2.31 (1.38–3.86)	2.04 (1.13–3.69)	0.02
CRP > 150 mg/dL	3.54 (1.83–6.86)	2.00 (0.88–4.57)	0.01
Blood leukocyte count **	1.56 (1.02–2.37)	0.81 (0.60–1.10)	0.17
CSF leukocytes < 4 cells/mm3	Reference	–	
CSF leukocytes 4 to 100 cells/mm3	1.33 (0.83–2.12)	–	
CSF leukocytes 100 to 1000 cells/mm3	1.31 (0.69–2.49)	–	
CSF leukocytes > 1000 cells/mm3	1.17 (0.53–2.55)	–	
CSF protein > 0.60	2.39 (1.57–3.65)	2.29 (1.40–3.75)	< 0.001
CNS infection	Reference	–	
CNS inflammatory disease	0.69 (0.31–1.5)	–	
Systemic infection	1.00 (0.59–1.72)	–	
Other neurological disease	0.70 (0.42–1.18)	–	
Non-neurological non-infectious disease	0.46 (0.11–1.99)	–	

The multivariable analysis used an imputed dataset with 5 imputation rounds**,** all variables in the table were entered in the multivariable logistic regression model simultaneously. Abbreviations: GCS = Glasgow Coma Scale; CRP = C-reactive protein; CSF = Cerebrospinal fluid; CNS = Central Nervous System.

The group of CNS inflammatory diseases consisted of 113 of 1152 episodes (10%). The rate of unfavourable outcome differed between the definitive diagnoses included in this category. Eight out of ten (80%) episodes with confirmed autoimmune encephalitis (AE) had an unfavourable outcome, 18 out of 26 (69%) with possible AE of unknown cause, eight out of nine (89%) with myelitis, and 32 out of 43 (74%) with other neurological autoimmune disorders (Table 2). Unfavourable outcome was due to residual neurological sequelae in 62 out of 74 (84%).Twenty-three of 113 episodes (20%) with CNS inflammatory disease were initially treated with antibiotics consisting of amoxicillin and ceftriaxone according to bacterial meningitis protocol. Aciclovir was given in 35 episodes (31%). When probable CNS inflammation was diagnosed, first line immunosuppressive therapy (e.g., methylprednisolone (MPS), prednisone, intravenous immunoglobulins [IVIg]), was started in 87 of 113 episodes (77%) and escalation to second-line therapy (e.g., plasma exchange [PLEX], azathioprine, rituximab, cyclophosphamide, and mycophenolate mofetil [MMF], methotrexate) was required in 31 of 87 episodes (36%). First line therapy was commenced during initial admission, in 71 of 87 episodes (82%), with escalation to 2nd line therapy during this admission in 19 of 71 episodes (27%). Escalation to 2nd line therapy at a later point in the outpatient clinic or when readmitted was done in 10 of 71 episodes (14%). For 16 of 87 episodes (18%), first line treatment was only started after admission with escalation to 2^nd^ line immunosuppressive therapy in 2 of 16 episodes (13%).

The time between presentation to immunosuppressive treatment was known in 84 of 87 (96%), with a median time to treatment of 5 days (IQR 1- 30). A univariate analysis for time to treatment and outcome showed no association (odds ratio 0.83 [0.51–1.35], *P* = 0.45). Immunosuppressive treatment was not administered in the remaining 26 episodes for various reasons, including spontaneous recovery occurred in 6 episodes (26%), mild symptoms well-manageable with symptom relief medication in 6 episodes (26%), a self-limiting disorder in 4 (15%), and one patient died before commencing immunosuppressants (4%).

## Discussion

Our study shows that patients presenting with an episode of suspected CNS infection have a high risk (36%) of experiencing an unfavourable outcome. Consistent with previous studies, advanced age was found to be an independent predictor of unfavourable outcome^12–14^. The association between outcome and focal cerebral deficits, an altered mental state, and elevated CSF protein count is likely to reflect the severity of neurological damage, while thrombocytopenia and tachycardia are associated with sepsis^15–19^.

Patients who were eventually diagnosed with CNS inflammatory disease showed the poorest prognosis. This association can be explained by various factors, including the severity of the conditions. Unfavourable outcome was most prevalent in confirmed cases with autoimmune encephalitis (80%) or suspected autoimmune encephalitis (69%). These rates are relatively high compared to previous studies on autoimmune encephalitis, which reported rates ranging from 13 to 80%, depending on the follow-up duration, associated antibodies, and aetiology of the autoimmune encephalitis episode^20–26^. The difference in outcome between our cohort and the literature may be due to the limited follow-up time in our study, as most studies provided an extensive follow-up time of up to 33 months, with outcomes that continued to improve for up to 18 months after symptom onset^21,22,25,27^. Moreover, our cohort consisted of a relatively small group of autoimmune encephalitis cases, most of whom were admitted to a tertiary hospital. Furthermore, our observation that other inflammatory conditions, like inflammatory myelitis, vasculitis, Guillain Barre syndrome, neurosarcoidosis, are associated with an unfavourable outcome aligns with existing literature^28–31^.

Contrary to previous studies on predictors for unfavourable outcome in CNS infections, the presence of seizures or an immunocompromised state, e.g., diabetes mellitus, did not show an association in our cohort^32,33^. This can be explained due to the heterogeneity in diagnoses in the cohort. Notably, for these variables, the odds ratios shifted from indicating a higher likelihood to suggesting a lower likelihood of an unfavourable outcome between the univariate and the multivariate analyses. This change could be caused by interactions with a covariate, such as final diagnosis associated with diabetes or an immunocompromised state, although this is speculative.

In CNS inflammatory diseases, treatment choice frequently rely on expert opinions rather than on randomized controlled trials for comparing treatments. Although our study did not find an association between treatment delay and outcome in CNS inflammatory episodes, it is generally accepted that time to treatment is a modifiable risk factor for poor outcome. Moreover, accumulating evidence and recent guidelines point to the beneficial effects of early diagnosis and treatment on outcome^34–39^.

Currently, diagnostic methods only establish the etiologic cause in 50% of encephalitis cases, with at least 10% being diagnosed as autoimmune encephalitis, of which causative anti-neuronal antibodies could only be detected in 35%^1,26,40^. The median time to treatment initiation for a CNS inflammatory disease was 5 days, and treatment was started only after 30 days in 25% of the cases. This can be attributed to an insidious onset of the disease, as well as the lengthy duration of diagnostic tests for autoimmune encephalitis, such as anti-neuronal antibody testing. Such episodes can initially be suspected of infectious meningoencephalitis, but after microbiological tests return negative, diagnostic tests for autoimmune encephalitis are ordered and generally take several weeks to generate results. Unfortunately, empirical treatment for autoimmune disorders is often not initiated while waiting for these tests^41^.

Prompt immunotherapy has been associated with a favourable outcome for all types of autoimmune encephalitis, as spontaneous clinical improvement is infrequent^21^. Various treatment options are available, including corticosteroids, TPE, IVIG, and immunosuppressant drugs. Treatment choice depends on the pathophysiology of the specific type of autoimmune encephalitis and the patients’ comorbidity^26,42^. A recent study concluded that more aggressive treatment regimens in autoimmune encephalitis patients improved the 2-year outcome. However, a comment on this study suggested that first-line immunotherapy's effect was underestimated while second-line immunotherapy's effect was overestimated^26,43^. Based on our study, early treatment with anti-inflammatory drugs should be considered to minimize the risk of an unfavourable outcome in cases of CNS inflammatory diseases.

Our study had several limitations. First, episodes could only be included when a lumbar puncture was performed, and the researchers identified the patients. This may have resulted in missed inclusions. Second, in some episodes, the final diagnosis was based on the clinical picture rather than microbiological evidence, demonstrated antibodies or radiological features, and thus may have led to misclassification. To solve this, we scored the final clinical diagnoses with two independent investigators and a third to solve discrepancies representing a proper classification process. Third, patients were predominantly admitted to a tertiary hospital and were inherently more complex than those in a general hospital, potentially causing selection bias. However, the majority of patients presented at the emergency department, reducing this risk of bias. Fourth, we did not analyse predictors for outcome for each diagnostic category separately. Instead, our focus was on evaluating all adults presenting with a suspected CNS infection, aiming to aid physicians in the acute setting, particularly when patients are still undifferentiated. This approach allowed us to gain insights into which patient subgroup requires more targeted investigation on diagnostics and treatment in future research.

In conclusion, patients suspected of having a CNS infection are at high risk of experiencing an unfavourable outcome, stressing the urgent need for improving rapid and accurate diagnostics. Amongst this suspected CNS infection group, those eventually diagnosed with CNS inflammatory disease have the highest risk of an unfavourable outcome. Our findings underscore the importance of prioritizing diagnostic and treatment improvements in this population. Based on our study, early treatment with immunosuppressive drugs may be considered to reduce the risk of an unfavourable outcome in cases of CNS inflammatory diseases.

## Data Availability

Anonymized data not published within this article will be made available by request from any qualified investigator. Proposals can be directed to the corresponding author, Matthijs Brouwer, by sending an email to ipace@amc.nl.
